# Biodiversity and disease risk in an algal biofuel system: An experimental test in outdoor ponds using a before-after-control-impact (BACI) design

**DOI:** 10.1371/journal.pone.0267674

**Published:** 2022-04-28

**Authors:** Spenser L. Widin, Kia M. Billings, John McGowen, Bradley J. Cardinale

**Affiliations:** 1 School for Environment and Sustainability, University of Michigan, Ann Arbor, Michigan, United States of America; 2 Arizona Center for Algae Technology and Innovation (AzCATI), Arizona State University, Mesa, AZ, United States of America; 3 Department of Ecosystem Science and Management, Penn State University, University Park, PA, United States of America; Zhengzhou University, CHINA

## Abstract

For outdoor cultivation of algal feedstocks to become a commercially viable and sustainable option for biofuel production, algal cultivation must maintain high yields and temporal stability in environmentally variable outdoor ponds. One of the main challenges is mitigating disease outbreaks that leads to culture crashes. Drawing on predictions from the ‘dilution effect’ hypothesis, in which increased biodiversity is thought to reduce disease risk in a community, a teste of whether algal polycultures would reduce disease risk and improve feedstock production efficiencies compared to monocultures was performed. While the positive benefits of biodiversity on disease risk have been demonstrated in various systems, to the best of our knowledge this is the first test in an algal biofuel system. Here, the results a before-after-control-impact (BACI) experimental design to compare mean monoculture (control) and polyculture (impact) yield, stability, and productivity before and after fungal infection when grown in 400-L outdoor raceway ponds are presented. It has been found that polycultures did not experience a reduction in disease risk compared to monocultures or differ in production efficiencies throughout the course of the 43-day experiment. These results show that polyculture feedstocks can maintain similar levels of productivity, stability, and disease resistance to that of a monoculture. Determining whether these results are generalizable or represent one case study requires additional outdoor experiments using a larger variety of host and pathogen species.

## Introduction

Over the last several decades, renewable microalgal derived biofuels have begun to show increased promise as a replacement for petroleum-based transportation fuels as a way to curb global CO_2_ emissions. Compared to conventional terrestrial crops (e.g. corn, switchgrass, oil palm) microalgal feedstocks have potential to achieve higher lipid yields per unit area than conventional crops without the need for arable land that could be used for food production [1–4]. Like terrestrial crop production, the predominant approach to large scale cultivation of algae has largely focused on genetic engineering and strain selection to identify single species that maximize lipid production when grown in monoculture under laboratory settings [1, 4, 5]. However, the high yields achieved by genetically or strain selected species in the lab rarely hold under field conditions, such as in large-scale open pond outdoor raceways that are the most economically viable method for generating large feedstock quantities [1, 6]. Indeed, algal feedstocks grown in outdoor raceway ponds have proven more difficult to maintain because of invasion by unwanted species of competing algae or cyanobacteria [7], invasion by herbivores that consume the crop [8], invasion by parasites and diseases that kill the focal algae [6] or environmental conditions that fluctuate beyond the tolerance limits of the focal species [9].

In response to the challenges of algal monoculture production, particularly in outdoor open raceway ponds, researchers have begun to consider how ecological engineering of diverse algal feedstocks can alleviate the challenges faced by monoculture feedstock production [10, 11]. Several studies have shown that when compared to the average monoculture, algal polycultures composed of multiple species can be designed to improve the total production of biomass, the temporal stability of feedstock production in variable environments, and desirable biochemical properties of feedstocks that are upgraded to biocrude [12–16]. For example, Shurin *et al*. [14], found that particular polycultures were able to achieve high algal biomass yield than their most productive monocultures. Similarly, diverse algal feedstocks have shown to be on par or exceed nutrient use efficiencies of the average monocultures [13, 17, 18]. Additionally, Godwin *et al*. [17] demonstrated that biodiverse algal cultures were able to maintain higher levels of multifunctionality (simultaneously maintain high levels of yield, stability, invasion resistance, etc.) than any of the component species grown in monoculture. However, it is important to note that most of the aforementioned studies have been performed in laboratory settings. As such, it remains unclear whether the benefits that are sometimes conferred by biodiversity will hold under commercial scale outdoor cultivation where the risk of pests, parasites, and pathogens are more substantial than they are in the safety of the lab [6, 19–21].

One of the greatest risks to outdoor cultivation of algal feedstocks is their susceptibility to invasion by unwanted pathogens, particularly pathogens such as chytrid (phylum Cryptomycota) and aphelid (phylum Aphelida) fungi [6, 22]. These fungal pathogens penetrate host cells, consume intracellular contents so as to produce large quantities propagules that are released through the ruptured host cell wall, causing host cell mortality [23]. As they do so, the fungi are able to quickly proliferate through algal cultures, often leading to culture crash and complete loss of feedstock productivity [22]. Currently, two main strategies are used to mitigate the impact of infection. One involves a salvage harvest of the culture, followed by subsequent disinfection of the culture tanks, prior to reestablishing the outdoor pond operation [6]. This approach leads to yield reductions in addition to increased operational costs, making the strategy economically infeasible. A second strategy of fungal control involves the application of chemical fungicides to reduce the risk of disease spread [6, 10, 24, 25]. While chemical fungicides have proven to be effective in the short term, they can be expensive, have the potential for target species to develop resistance, can pose certain human health risks, and can have unintended environmental impacts [26, 27]. Given these limitations, there has been recent interest in developing better methods of fungal control in algal feedstock systems [28, 29].

A recently proposed method for fungal control utilizes a concept from the field of Disease Ecology called the “dilution effect” [29]. The dilution effect occurs when an increase in host diversity leads to a reduction in the risk of disease within a community [30, 31]. One proposed mechanism for disease dilution occurs when less susceptible host species reduce the abundance of more susceptible host through interspecific competition [32]. That is, the presence of less susceptible hosts ‘dilute’ the risk of disease establishment and spread throughout the community. If the dilution effect were to operate in algal feedstocks, then it might be possible to ecologically engineer the composition of feedstocks to have species that have differential fungal resistance, niche complementarity, and high measures of productivity (e.g. temporal stability, yield, etc.). In turn, one might be able to simultaneously safeguard against disease outbreaks while enhancing feedstock yields through time [29]. But while many studies have shown the operation of dilution effects in natural systems, the idea has not been tested using algal feedstock cultivation [33, 34].

In this study, the question of whether ecologically engineered multi-species consortia of algae would be more resistant to crop failure caused by fungal pathogens than single species monocultures is addressed. It is hypothesized that due to a dilution effect, ecologically engineered algal polycultures would maintain higher measures of feedstock production compared to monocultures of the most productive species when both were simultaneously exposed to a fungal pathogen. This hypothesis was tested by conducting a statistically rigorous, well replicated BACI experiment (before vs. after fungal infection, monocultures as controls vs. polycultures as the impact treatment) in outdoor raceway ponds. Algal monocultures and polycultures were grown in replicate raceways and were subjected to routine harvests, intentionally challenged with a fungal pathogen, and sampled regularly to estimate feedstock production metrics and fungal infection.

## Materials and methods

### Microalgal species selection

As the goal of this experiment was to not to simply test whether any multi-species consortia could buffer against disease risk, but rather whether the best algal feedstock monoculture and multi-species consortia, would differ in terms of productivity metrics in outdoor ponds in response to disease, results from prior experiments were used to determine the model algal species. From a three-phase series of prior experiments the best monoculture and multi-species consortia were identified. Below details of the following three phases are outlined: I) experimental biculture comparisons of yield from 55 species pool, II) laboratory comparison of yield, temporal stability, and nutrient use efficiency monocultures and polycultures best six species from Phase I in laboratory, and III) field comparison of yield, stability, and pest invasability between single best monoculture and polyculture from Phase II in field experiment.

Phase I involved 55 species of freshwater green algae that are included in the U.S. Department of Energy’s Aquatic Species Program and were identified in U.S. EPA’s 2007 National Lakes Assessment as being the most widespread and abundant species across North America (so they would not pose a risk to natural habitats in the event of a release). Using these species, an initial set of laboratory experiments compared the yield of species bicultures to that of monocultures to screen for increased biomass production in cultures with co-occurring algal species [35]. Results from this work lead to the selection of six species that were found to routinely be involved in increased biomass production of algal polycultures. During Phase II, laboratory experiments were used to evaluate whether polycultures of these six species would increase biomass production and temporal stability compared to each species grown alone in monoculture [16, 17, 36]. Phase III transitioned to field-based experiments in which the single best monoculture and polyculture consortia from Phase II were compared for production of algal biomass and biocrude yield, temporal stability, risk of invasive algae, potential for culture crash, and ability to maintain more of these functions at higher levels [12]. Based on the results of Phases I-III the single best monoculture (*Selenastrum capricornutum*) identified for algal feedstocks from prior work, and single best algal polyculture (*Selenastrum capricornutum*, *Chlorella sorokiniana*, and *Scenedesmus obliquus*) were selected to pit against each other in outdoor raceway cultivation that is more realistic to that of commercial scale production.

### Experimental units

**Fig 1:**
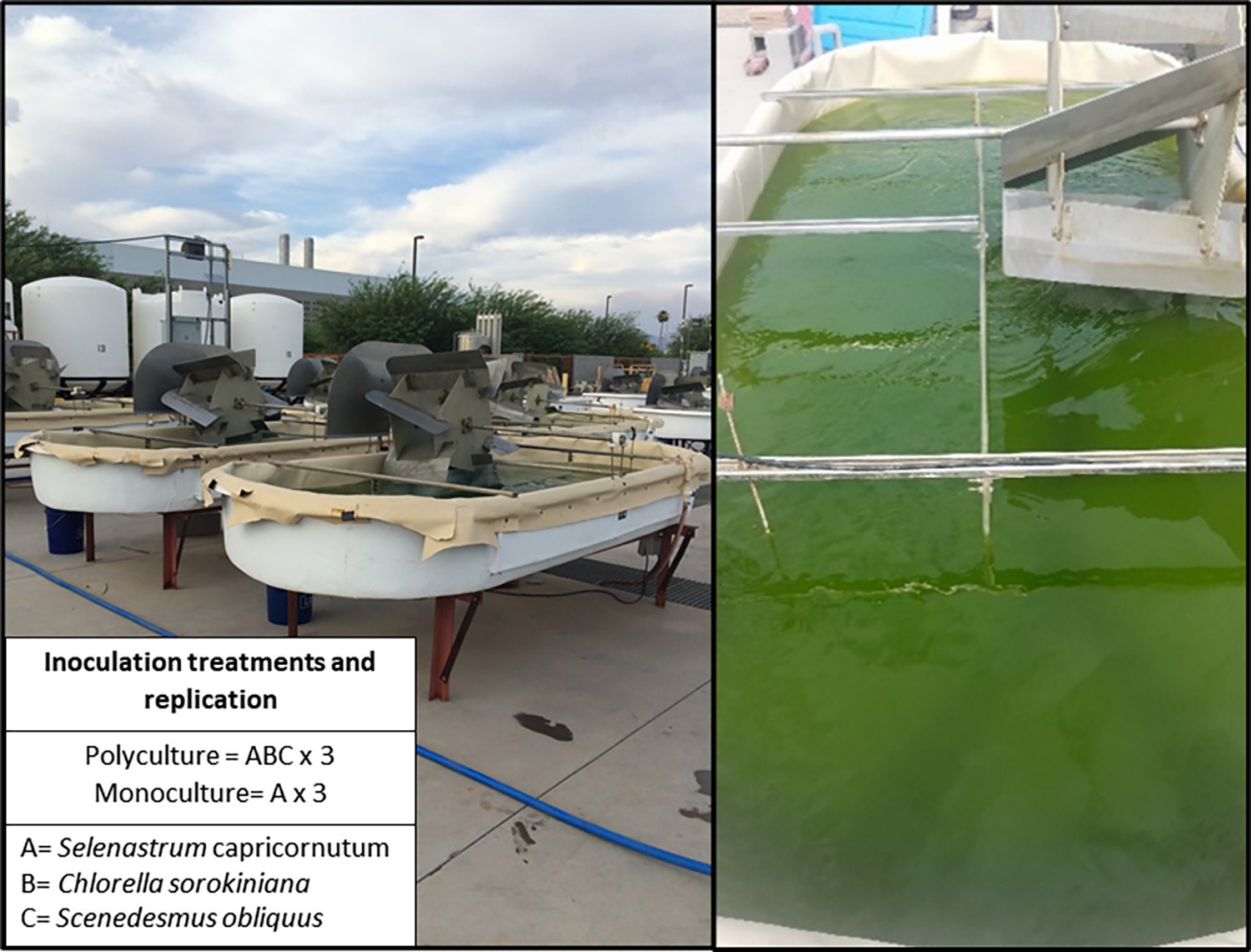
Experimental set-up of outdoor cultivation ponds. Photographs of 400-L experimental outdoor raceways located at Arizona Center for Algal Technology and Innovation (AzCATI) in Mesa, Arizona. Inset text summarizes experimental design.

The study site was located at the Arizona Center for Algae Technology and Innovation (AzCATI) at Arizona State University’s Polytechnic campus in Mesa, AZ (Fig 1). Six identical raceway ponds measuring 3.5m by 1.5m (approximately 4.2m^2^ surface area) filled to a depth of 10cm to produce a 400-L capacity were utlized. Each pond at AzCATI was equipped with a mechanical paddle wheel to constantly mix the algal cultures (flow rate of 9.3 cm s^-1^), an air stone to bubble CO_2_ into solution (5 L min^-1^ based on measured pH so that a constant pH could be maintained), and a YSI 5200A-DC (YSI Inc., Yellow Springs, OH, USA) probe to measured physical and chemical conditions of the ponds (temperature, salinity, conductivity, dissolved oxygen, oxygen saturation, and pH) at 15 minute intervals. Each 400-L raceway was initiated with the 2 time the standard concentration of constituents of BG-11 growth media [37], which is a common media for growth of algal feedstocks. As will be shown (*Section Physical and chemical conditions of raceways*) physical and chemical conditions among replicates were statistically similar between Control-monoculture and Impact-polyculture treatments.

### Experimental design

This experiment used a BACI design (Before, After, Control, Impact) to determine if algal polycultures (the ‘Impact’ group) increase the production and stability of algal feedstock relative to algal monocultures (the ‘Control’ group) when they are exposed to an infectious fungal parasite (‘Before’ vs. ‘After’ infection). The Control treatment consisted of a monoculture of *S*. *capricornutum*, while the Impact treatment consisted of a polyculture of *S*. *capricornutum*, *C*. *sorokiniana*, and *S*. *obliquus* (Fig 1). The Control and Impact treatments were each replicated in 3 raceway ponds.

Prior to their inoculation in the 400-L raceway ponds, each algal species was scaled up individually in laboratory cultures, first in 800-mL sterile glass columns containing BG-11 growth media, which were then transferred into 15-L plexiglass panels containing BG-11 growth media that were exposed to continuous light and a constant delivery of gaseous CO_2_ (S2 Fig in S1 Appendix). Once adequate cell densities were reached in laboratory cultures, each of the 400-L raceways was inoculated with the same starting concentration of dry algal biomass (0.084 g L^-1^). This resulted in 0.084 g L^-1^ of *S*. *capricornutum* being added to each of the three Control (monoculture) raceway ponds, and 0.028 g L^-1^ of each of the three species into the Treatment (polyculture) raceway ponds.

The Before and After portion of the study represented sampling periods before and after a fungal parasite was intentionally introduced to the ponds. Raceway ponds were monitored for 23 days before a fungal parasite was introduced (sampling is described later in *Section Experimental design*). During this time period, through microscopic examination of algal samples there was no significant sign of fungal infection detected in any of the raceway ponds. Then, on day 23 and again on day 25, each raceway pond was inoculated at less than 1% of total cell density with 75mL of *Desmodesmus armatus* from a nearby outdoor pond that was nearly 100% infected with a local fungal parasite that is a common pest at AzCATI (see *Results* for description of fungal parasite). The fungal parasite was intentionally introduced using a non-focal species of algae because (1) cultures of infected *D*. *armatus* are routinely maintained at AzCATI for study of the fungus, making it a readily available and controlled source of infection, and (2) *D*. *armatus* is a morphologically distinct, competitively inferior species (based on prior field experience and observations) that can be used as a ‘tracer’. As a tracer species an equal amount of infection to all ponds was able to be introduced, after which, the ponds were monitored to show that the source of infection (*D*. *armatus*) was successful at initiating disease in other algal species; yet *D*. *armatus* did not establish itself in the ponds or alter the intended species composition of algae (as will be discussed later).

On day 27, five days after the introduction of the fungus, each raceway pond was dosed with 1-ppm of Secure ® with the active ingredient fluazinam, which is a pesticide that is commonly used to control fungal pathogens during algal feedstock cultivation at AzCATI (U.S. Patent Application No. 14/351,540; Publication No. 20140378513; Published Dec. 25, 2014; Sapphire Energy Inc., Applicant). By applying a pesticide that is normally used to control fungal proliferation during feedstock production, this study was intended to test whether algal polycultures offer any additional benefits for pest control above and beyond the traditional controls that are applied during feedstock growth. For the remaining 20 days after fungal infection (After period), the raceways were sampled and harvested utilizing the same methods as during the Before period (described next).

### Raceway sampling and processing

Following the initial inoculation of algae in the raceway ponds, samples were collected from each pond every 2-days using a sterile 50-mL polypropylene centrifuge tubes by submerging the tubes in same well mixed portion of each raceway. From these samples, two sub-samples were taken. One 15-mL subsample was used to determine daily estimates of feedstock biomass by measuring ash free dry weight (AFDW). AFDW was measured utilizing the methodology described in McGowen et al. [38], which involved vacuum filtering the 15-mL subsample through a pre-weighed 0.2μm glass microfiber filter, dried at 105°C, ashed at 500°C leaving an inorganic compound residue and then re-weighed. The difference in weight before and after ashing gives AFDW in g of biomass L^-1^. For each sample, three separate measures of AFDW were averaged to produce one estimate per sampling event. A second 1-mL subsample was fixed with a 1% phosphate-buffered formalin solution to preserve the sample for quantification of algal cell densities and the proportion of cells with fungal infection. This was achieved by performing manual counts of individual species cell density (healthy and infected cells) using a hemocytometer and light microscopy. Following each sampling event, fresh water was added to the raceway ponds to maintain their original volumes at 400-L and compensate for evaporative losses.

In addition to sampling the ponds every 2-days, the feedstock of algae in each raceway pond was harvested weekly to measure areal productivity. Harvesting was performed using a sump pump placed directly into the raceway while the raceway was actively mixed by the mechanical paddle wheel. With exception of the first harvest, for which only 70% of the volume was removed due to the fact that biomass was still increasing, all subsequent harvests were performed by pumping out 90% of the volume of the raceways, which is a more standard harvest. Immediately after each harvest, raceways were filled back to their starting volumes of 400-L with fresh water replenished with nutrients to produce the same initial concentrations of BG-11 growth media.

### Data analysis

The aforementioned data collection was used (*Section Experimental design*) to calculate four response variables for each pond–areal productivity of feedstock, the stability of feedstock biomass through time, maximum biomass yield achieved immediately prior to harvest, and the proportion of algal cells infected with fungus. Areal productivity is a measure of the average daily biomass production (g of Biomass m^-2^ day^-1^) based on the surface area of each raceway pond. To calculate areal productivity, the average daily volumetric AFDW was converted to the mean daily areal productivity by dividing by 4.2 m^2^ (the surface area of the 400-L raceways). Average daily productivity of each raceway was calculated for the time frame between harvests, which resulted in two average daily productivity measures of each raceway for the Before fungal infection period and two measures for the After period.

Temporal stability of feedstock biomass was quantified by calculating the inverse of the coefficient of variation (CV) as the mean divided by standard deviation for daily AFDW (g biomass L^-1^ day^-1^) for each growth phase between harvests. To quantify the overall stability for each treatment (Control-mono vs. Impact-polyculture), individual estimates of the CV^-1^ for growth phases within each period (Before vs. After infection) were average. This allowed for examination of whether there were any significant differences in stability of daily biomass production between Treatments (control vs. impact) and Periods (before vs. after fungal infection).

The maximum biomass yield achieved in a raceway pond was taken to be the peak algal biomass (g biomass L^-1^) achieved immediately before harvesting each raceway. Samples taken immediately before each harvest event were used to calculate AFDW, providing an estimate of total amount of biomass by volume produced between each harvest event. Using these measurements, any differences between Treatments (control-mono vs. impact-polyculture) and Periods before vs. after fungal infection in the average maximum biomass yield achieved between harvest were able to be determined.

Fungal infection and community composition (polycultures only) were measured to evaluate any difference in response to infection between treatments. Fungal infections were quantified by calculating the proportion of algal cells infected with the fungal parasite for each species (the total healthy and infected cell density divided by the density of infected cells) for each sampling event to plot a time-series of infection proportions. Algal cells were categorized as infected if they had any of the following signs of infection: fungal zoospore attached to the cell wall, cellular contents displaced by the growing parasite, and/or any remaining unconsumable cellular contents of the host algal cell called a residual body (S1 Fig in S1 Appendix) [22, 39, 40]. Community composition for the Impact treatment was quantified as the proportion of total cell density represented by each algal species. When calculating the four response variables, data collected prior to the initial 70% harvest on day 8 were not used so that comparisons between each subsequent harvest represent a response in the algal cultures to the same magnitude of a routine disturbance (i.e., 90% harvest). In addition, data collected between infection (day 23) and the first post infection harvest (day 30) were not used in the analysis. These data were excluded because the fungal pathogen was initially introduced 2 days after a harvest which allowed the algal cultures to experience 2 days of growth without the potential stress of infection. Including this particular set of dates (days 23–30) in the After-infection period would include a portion of the culture’s response to harvest without stress from a fungal pathogen, which is more characteristic of the Before-infection period. Thus, to ensure that the data included in the After-infection period is accurately indicative of the response of infected algal cultures to routine harvesting, measurements from the first sampling event after the day 30 harvest were only included.

#### BACI analysis

A BACI analyses was performed to determine if the four aforementioned response variables (*Section Raceway sampling and processing*) showed significant differences between the Control (monoculture) and Impact (polyculture) treatments, as well as the response of those two treatments to introduction of the parasitic fungus. The BACI approach described in Smith et al. [41]was used, which involved running linear mixed models for each of the response variables with treatment (Control—monoculture vs. Impact—polyculture), infection period (Before introduction of fungus vs After infection), and Treatment × Period interaction as fixed effects, and individual raceways as a random effect to account for random variation among experiment units (“lmer function” within R package lmerTest). To examine whether fungal infection has a differential effect between Treatments (control-monoculture vs. impact-polyculture), results of the linear models were subjected to an analysis of variance (ANOVA). A significant Treatment × Period interaction for each response variable was taken as evidence that each Treatment responded differently to infection from a fungal pathogen. These tests, in conjunction with the time-series estimates of infection proportions and community composition, provided insight into the biological cause for differential response in the various algal production metrics between treatments.

In addition to using BACI to compare the biological response variables, similar analyses were performed that compared the physical and chemical conditions of the raceway ponds to confirm homogeneity of conditions between treatments (See Supplemental Information). No data transformations were needed as assumptions of homogeneity of variance and approximate normality were met.

## Results

**Fig 2:**
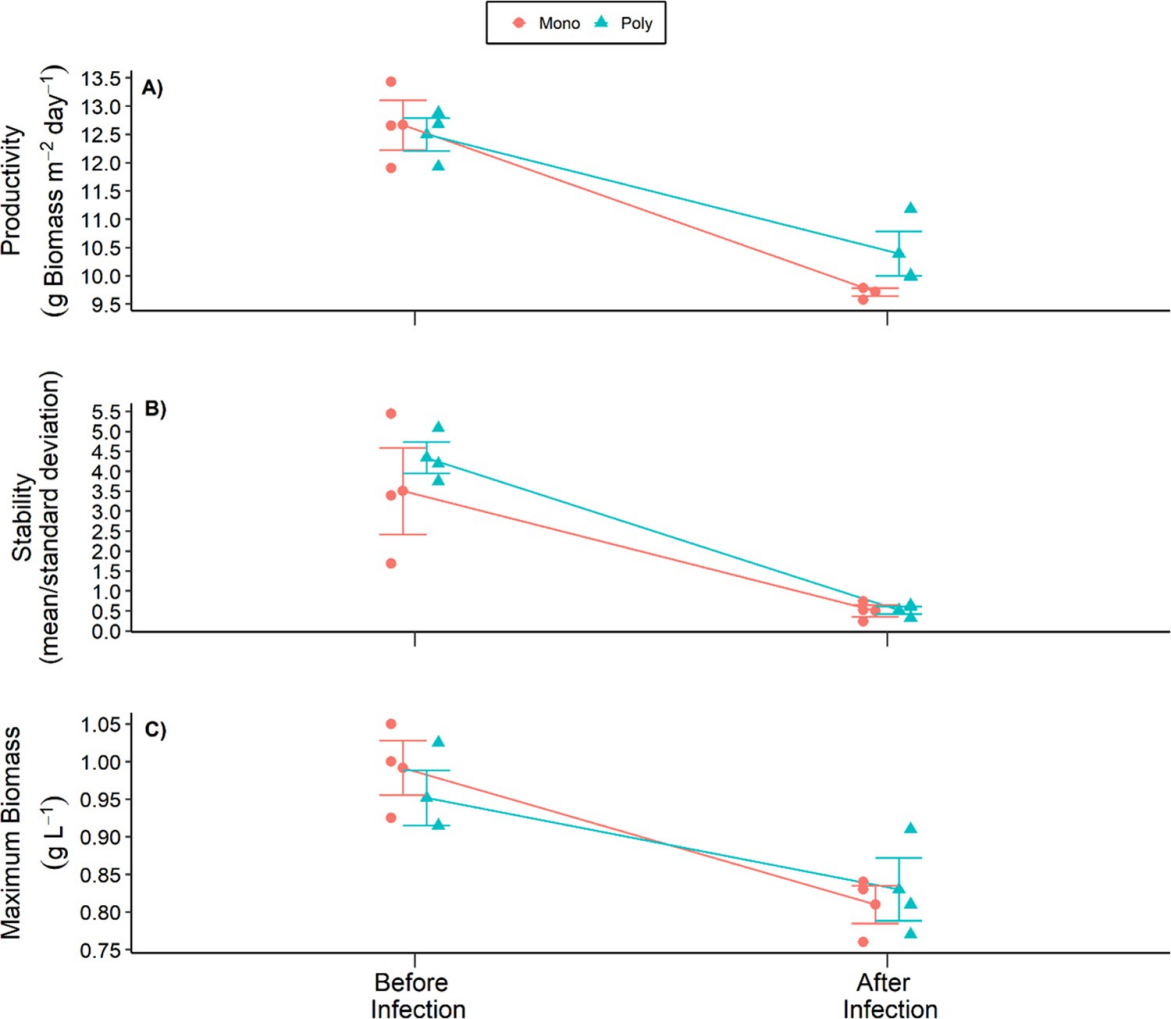
Mean production metrics of algal cultures before and after infection. Mean values for productivity (A), stability (B), and maximum biomass achieved immediately prior to harvest (C). Central data points with error bars show the grand mean of all replicates for each treatment with standard error bars. Offset data points represent the mean values for each replicate pond.

Physical and chemical conditions among replicates were statistically similar (*Section Physical and chemical conditions of raceways*) thus these results can reasonably be attributed to differences in biodiversity among treatments (control-monoculture vs. impact-polyculture). As expected, yield, productivity, and temporal stability across both treatments were reduced following fungal infection (Fig 2). Although we did not have replicate ponds that were uninfected with the fungus to definitively show that fungal infection was the cause of the decline in production metrics, this decline can reasonably be attributed to the fungal infection. This is because with the exception of a moderate increase in average water temperature (S3 Fig in S1 Appendix), there were no other biologically significant changes detected among the replicate ponds between the Before and After fungal infection periods (*Section Physical and chemical conditions of raceways*). Moreover, the small magnitude of difference in average water temperature is still within optimal temperature for algae production, and thus is unlikely the cause of such a drastic decrease in productivity [42]. Additionally, no significant effect of biodiversity on the response measured in feedstock production metrics to fungal infection was found (*Sections Areal productivity*, *Biomass stability*, and *Maximum biomass yield*). This can be seen in Fig 2, in which both before and after fungal infection there was no significant difference between monoculture and polyculture yield, productivity, and temporal stability. No difference between treatments is likely due to the species used for the monoculture treatment (*S*. *capricornutum*) being both competitively dominant and least susceptible to infection causing the polyculture treatment to rapidly become entirely composed of *S*. *capricornutum* (*Section* Fungal infection).

### Physical and chemical conditions of raceways

Over the 43-day course of the experiment, the mean physical and chemical conditions of the replicate ponds were comparable between treatments (Control-monoculture vs. Impact-polycultures) and between time periods (Before vs. After fungal infection). S3 Fig in S1 Appendix shows the time-series for average daily water pH, salinity, conductivity, oxygen saturation, dissolved oxygen, and water temperature. These time-series data are represented as treatment and time period means in S4 Fig in S1 Appendix, and a statistical comparison of treatment and period means is given in S1 and S2 Tables in S1 Appendix. S4 Fig in S1 Appendix and the two supplemental data tables in S1 Data show that the daily mean physical and chemical conditions of the ponds did change between time periods (e.g. water temperature increased, and oxygen saturation declined through time). However, there was no evidence that the physical/chemical conditions changed differently through time among treatments. Therefore, raceway ponds were homogenous with respect to each other in their physical/chemical conditions.

### Areal productivity

No significant difference in areal productivity, feedstock stability, or maximum biomass yield between the Control (monoculture) and Impact (polyculture) treatments was found. Areal productivity averaged 12.66 ± 0.44 g m^-2^ day^-1^ prior to infection in the Control (monoculture), and a nearly identical 12.50 ± 0.29 g m^-2^ day^-1^ in the Impact (polyculture) treatment (Fig 2A). After introduction of the parasitic fungus, areal productivity declined in both treatments to 9.72 ± 0.07 g m^-2^ day^-1^g in the Control (monoculture) and 10.39±0.40 g m^-2^ day^-1^ in the Impact (polyculture) treatment (Fig 2A). BACI analysis revealed no significant differences in areal productivity between treatments, and no difference in response of the treatments between the two time periods (Table 1). Thus, the means of the replicate ponds were the same among treatments and declined by the same magnitude after fungal infection.

**Table 1:** Analysis of variance effects test results.

		*Mean Productivity*			*Mean Stability*			*Mean Maximum Biomass*		
Effect	*df*	MS	*F*	*p*	MS	*F*	*p*	MS	*F*	*p*
Treatment	1	0.194	0.589	0.465	0.508	0.497	0.501	0.0003	0.080	0.785
Period	1	19.165	58.289	<0.000	34.919	34.1793	<0.000	0.069	18.290	0.003
Trmt:Period	1	0.531	1.6159	0.239	0.491	0.481	0.508	0.003	0.716	0.422

### Biomass stability

The mean feedstock biomass stability (inverse CV) prior to infection averaged 3.50 ± 1.09 for the Control (monoculture) and 4.34 ± 0.39 for the Impact (polyculture) treatments (Fig 2B). After fungal infection, mean feedstock stability decreased in both treatments to 0.50 ± 0.15 in the Control (monoculture) and 0.52 ± 0.10 in the Impact (polyculture) treatment (Fig 2B). BACI analysis showed no significant differences in either feedstock stability between treatments or response of the treatments between periods (Before vs. After infection). Therefore, the mean feedstock stability did not differ between treatments, and decreased by the same magnitude after fungal infection.

### Maximum biomass yield

The maximum biomass yield achieved prior to a harvest for the time-frame before fungal infection was 0.99 ± 0.04 g L^-1^ for the Control treatment, which was virtually equal to the 0.95 ± 0.04 g L^-1^ for the Impact treatment (Fig 2C). During the after-infection period, maximum biomass yield declined for both treatments to 0.81 ± 0.03 g L^-1^ for the Control verses 0.83 ± 0.04 g L^-1^ for the Impact treatment. The BACI analysis showed no significant difference in maximum biomass yield between treatments or a differential response to fungal infection (Table 1). Thus, both maximum biomass yield between treatments was the same for each period (Before vs. After infection) and decreased by the same magnitude after fungal infection.

### Fungal infection

Through microscopic examination (as described in *Section Data Analysis) D*. *armatus* successfully introduced the fungal pathogen, without becoming established in the cultures. Initial morphological characteristics identified the fungus as an Aphelida (class Aphelidea) [23]. Subsequent samples of infected algae from AzCATI, were cultured in the lab and later identified as *Ameobophelidium occidentale* using rDNA analysis. Since this fungi were from separate cultures outside of the experiment it is not certain that this is the same species, however the morphology and life cycle observed during the experiment are very similar to those identified for various Aphelida species [22, 23, 43]. Additionally, the *D*. *armatus* inoculum was from an open-air pond and could have contained other pathogenic agents. However, the dominant signs of disease within the cultures presented morphologically as Aphelid fungus in each of the algae species. This led to the belief that an Aphelid fungus was the main pathogenic stressor in the feedstock cultures.

**Fig 3:**
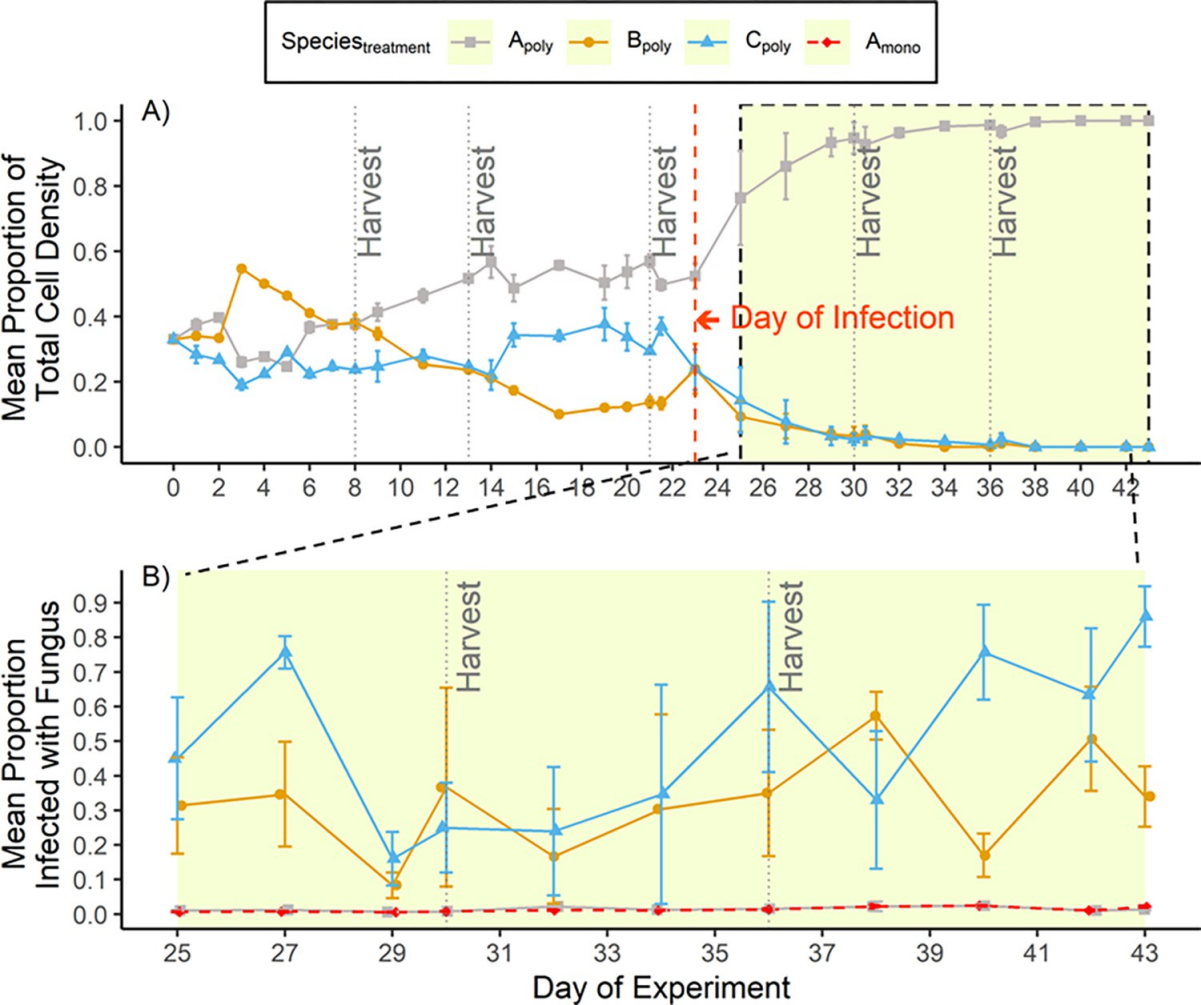
Time series of algal cell densities and infection metrics. Panel A shows the grand mean proportion of total cell density that is composed of each species in polyculture. Panel B shows the grand mean proportion of each species infected with the fungus in both polycultures and monocultures Bars represent the standard error. Highlighted yellow section of both graphs represents the experimental period post infection. Vertical light-grey dotted lines indicate harvest days. Data collected on harvest days are pre-harvest. Vertical red dashed line represents the day of infection (day 23). Infection proportions in panel B are only shown for the After Infection Period because no significant signs of infection were detected prior to day 24.

The lack of any differences in response variables between treatments (Control—vs. Impact) was most likely due to *S*. *capricornutum* being both a competitively dominant species and being least susceptible to the fungal parasite. Prior to the intentional introduction of the fungal parasite on day 23, no significant signs of infection were observed in any algal species (infection metric were calculate as the proportion of total cells showing signs infection, *2*.*5 Data Analysis)*. Prior to fungal infection, *S*. *capricornutum* maintained the highest proportion of total cell density in the Impact-polyculture treatments and *C*. *sorokiniana* and *S*. *obliquus* maintained a lower, but relatively similar proportional cell density to one another (Fig 3A). However, within five days of fungal infection, the average Impact (polyculture) treatment pond became dominated by *S*. *capricornutum* (>90% of total cell density), and these ponds were almost entirely composed of *S*. *capricornutum* by the end of the experiment (Fig 3A). *S*. *capricornutum* dominating the polyculture treatments after infection is likely attributed to *S*. *capricornutum* being least susceptible to the fungal pathogen and *C*. *sorokiniana* and *S*. *obliquus* both being highly susceptible. The average proportion of *S*. *capricornutum* becoming infected in either monoculture or polyculture was maintained at less than 0.03 (Fig 3B). In contrast, both *C*. *sorokiniana* and *S*. *obliquus* demonstrated high susceptibility to infection, and within 2 days after introducing fungus the proportion of cells infected of each species increased rapidly (Fig 3B). Throughout the remainder of the experiment C. *sorokiniana* and *S*. *obliquus* maintained high average infection proportions (*~*0.49 and ~0.32, respectively). The high susceptibility to infection of *C*. *sorokiniana*: and *S*. *obliquus* in conjunction with very low susceptibility of *S*. *capricornutum* likely explains why the Impact-polyculture treatments quickly became dominated by *S*. *sorokiniana*, in effect transitioning to a monoculture. Thus, this rapid shift in community composition within the Impact-polyculture treatment after infection reasonably accounts for Treatments (Control-monoculture vs. Impact-polyculture) not differing with respect to productivity, stability, and maximum biomass yield within the period After infection.

## Discussion

Many prior studies have shown that diverse algal cultures can enhance certain properties that are desirable in algal biofuel feedstock production. For example, polycultures relative to monoculture feedstocks have been shown improve temporal stability in variable temperature environments, delay invasion from unwanted algae species, and improve nitrogen and phosphorus use efficiencies [12, 16, 17]. Additionally, polycultures have demonstrated the ability to perform more functions at a higher levels compared to the component species grown in monoculture [12, 17]. However, to our knowledge, no study has tested whether diverse algal cultures help feedstocks resist the impacts of disease. Invasion from pest species in outdoor algal cultivation can rapidly result in culture crashes and complete loss of feedstocks, which presents one of the main limiting factors for successful cultivation [6, 20, 44]. Thus, developing effective disease mitigation strategies will improve the feasibility of large-scale algal biofuel cultivation.

Here the first empirical test of whether multi-species feedstocks increase resistance to a fungal pathogen is provided. Fungal pathogens, especially aphelid and chytrid fungal strains, represent one of the greatest challenges to algal cultivation, as they can quickly proliferate through cultures and result in high host mortality [22, 23]. This study hypothesized that more diverse algal cultures would reduce the prevalence of fungal disease by generating a dilution effect. The dilution effect occurs when an increase in host species diversity leads to a reduction in the risk of disease within an entire community [32]. Contrary to the hypothesis, it has been found that polycultures and monocultures did not differ in their disease risk, thus biodiversity did not confer greater disease resistance through a dilution effect. Because the competitively dominant species (*S*. *capricornutum*) was also the most resistant to the parasite, it quickly dominated the monocultures after infection.

The lack of a dilution effect in this study contrasts with a body of literature from the field of disease ecology that has shown that more diverse communities are often less susceptible to disease than are less diverse communities [28, 45–47]. Biodiversity can ‘dilute’ the prevalence of disease via several mechanisms. One proposed mechanism for disease dilution occurs when less susceptible host species reduce the relative abundance of more susceptible host species in a community [32]. In turn, a greater relative abundance of less susceptible host species ‘dilute’ the risk of disease establishment and spread throughout the community. In this study system, this mechanism did not operate because its assumptions were not met. To the contrary, it was found that the competitively dominant algae (*S*. *capricornutum*) was also least susceptible to fungal disease. Therefore, when *S*. *capricornutum* came to dominate the algal polycultures, it increased the relative abundance of less susceptible hosts, and decreased the relative abundance of more susceptible host species (*C*. *sorokiniana* and *S*. *obliquus*). In turn, there was no reduction in disease risk in the algal polycultures compared to a monoculture of *S*. *capricornutum* (Fig 3). Essentially, the polycultures behaved as a monoculture with disease resistance driven by the biology of *S*. *capricornutum*. It is possible that the application of pesticides contributed to the reduced biomass production in both polycultures and monocultures in the After-infection period. However, biomass production metrics began decreasing immediately following infection, prior to application of pesticides, suggesting that initial decline in biomass production was driven by fungal infection stress.

Of course, these results are limited in scope because of the limited species pool of algae and that they were exposed to just one type of pathogen. The species that were the focus of this study were chosen based on several years of prior work that specifically sought to identify the most productive and stable species monoculture (*S*. *capricornutum*) and polyculture from a species list that started with 55 Chlorophycean and Charophycean green algae that are commonly used in biofuel research [12, 16, 17, 35, 36]. The goal was to pit the single best mono- verses polyculture against each other in typical outdoor cultivation setting as a test of disease resistance to determine if species consortia have advantages over traditional monoculture feedstocks that are exposed to and treated for common fungal infections (i.e. with pesticides). However, because the species pool was limited, this experiment should be interpreted as a single case study, rather than as general evidence against the operation of dilution effects in algal feedstocks. It is quite possible, perhaps even likely, that dilution effects will be identified in future studies that use different species pools that conform more to the assumptions of models of dilution effects. Accordingly, it may still be possible to identify algal species that have complimentary levels of disease susceptibility spanning a range of pathogens that might improve temporal yields and stability in outdoor cultivation ponds.

In addition to finding no benefit of algal diversity for disease resistance, no evidence was found that algal polycultures outperform monocultures in terms of algal production, stability, and response to pesticides. Such results also contrast with certain prior studies, such as those by Shurin *et al*. [10, 14] and Stockenreiter *et al*. [15, 18], which have found that the best performing polyculture obtains higher biomass yields than that of the component species grown in monocultures. However, it is emphasized that most prior work has been performed in highly controlled laboratory conditions, and it is presently unclear if such results can be replicated in the less controlled, more variable conditions of outdoor production. These results are more comparable to those of Godwin *et al*. [12], who found that under outdoor cultivation, polycultures obtained lower biomass yield and stability compared to the best performing component species grown in monoculture. This one case study emphasizes that diverse algal polycultures can maintain similar levels of productivity and have equal disease resistance to that of certain monoculture. Whether or not this study represents a generality will require that additional outdoor pond experiments using a greater variety of species are performed.

## Supporting information

S1 Data Co-optima data. (XLSX)

S1 Appendix (DOCX)
