# Supplementary material for: Biodiversity and disease risk in an algal biofuel system: An experimental test in outdoor ponds using a before-after-control-impact (BACI) design
Source: PLoS One. 2022 Apr 28;17(4):e0267674. doi: 10.1371/journal.pone.0267674 (PMC9049517; doi:10.1371/journal.pone.0267674)
Supplement: S1 Appendix — (DOCX) [file pone.0267674.s002.docx]

**Appendix: Supplemental Figures and Statistical tables**

**
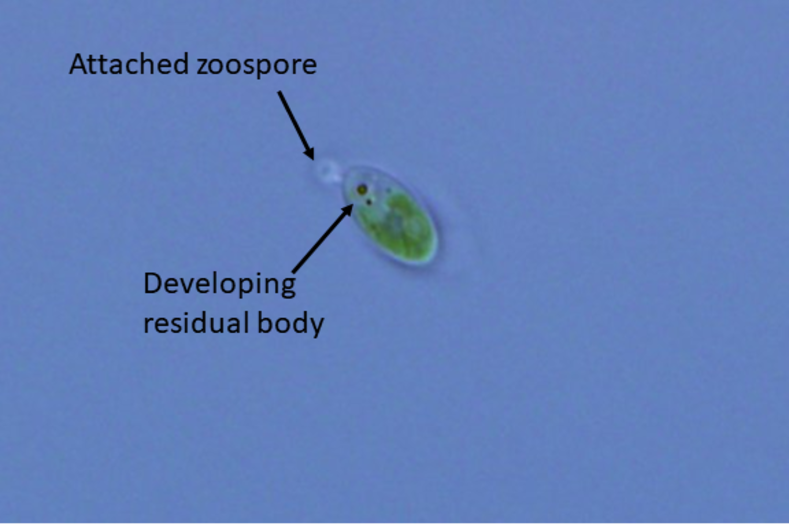
**

**Figure S1.** Compound microscope image at 400x of characteristic infection properties in *Scenedesmus obliquus*.

**
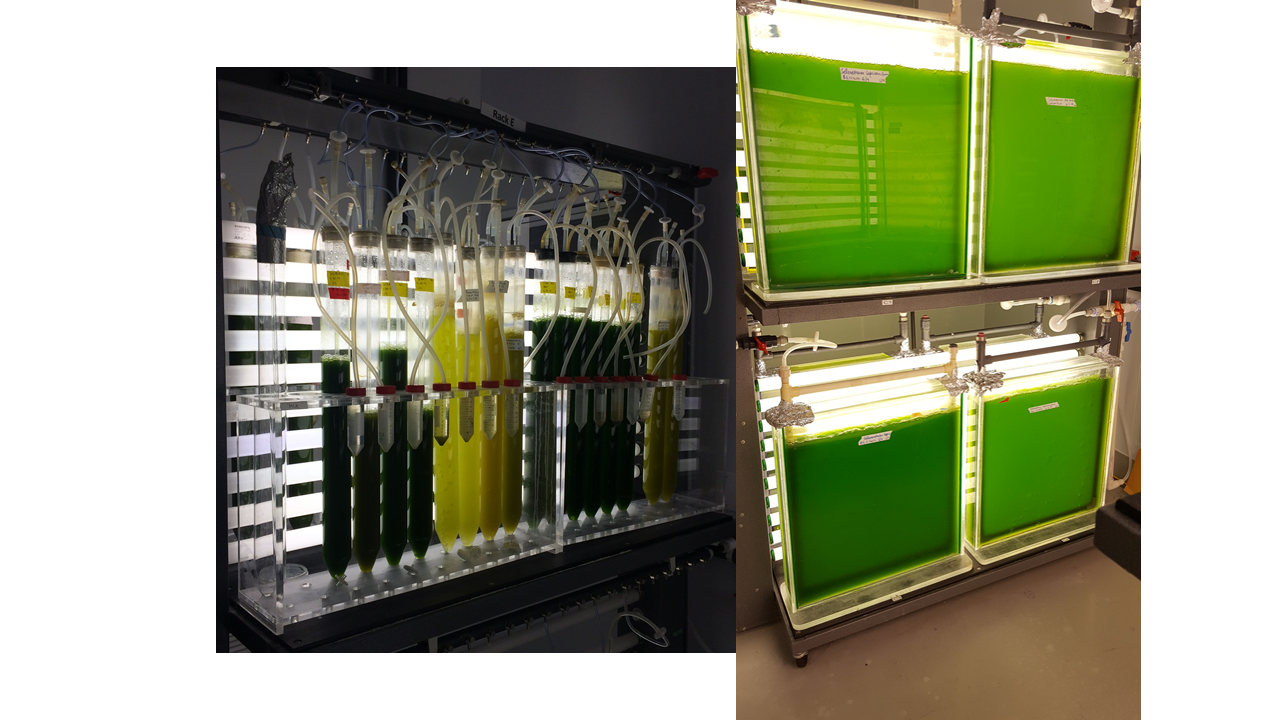
**

**Figure S2.** Photographs demonstrating the scale up process of the algal cultures prior to inoculation in large experimental raceways.


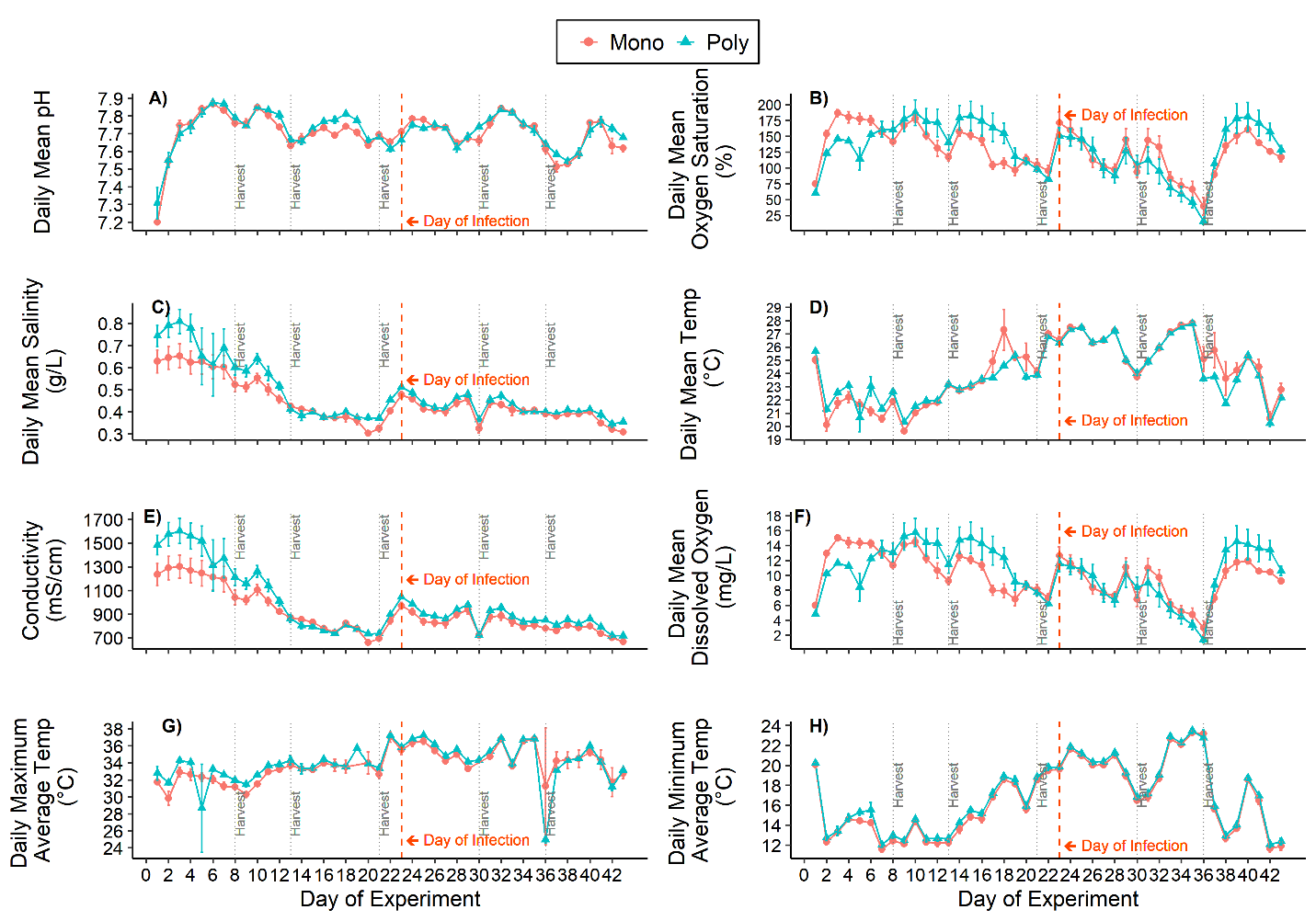


**Figure S3:** Mean daily measurements for physical and chemical conditions between ponds in each treatment. Vertical bars indicate standard error. Note: the large increase in the dissolved oxygen in the polyculture tanks following the last harvest is believed to be due to a faulty probe in one of the replicate tanks, thereby increasing the mean.


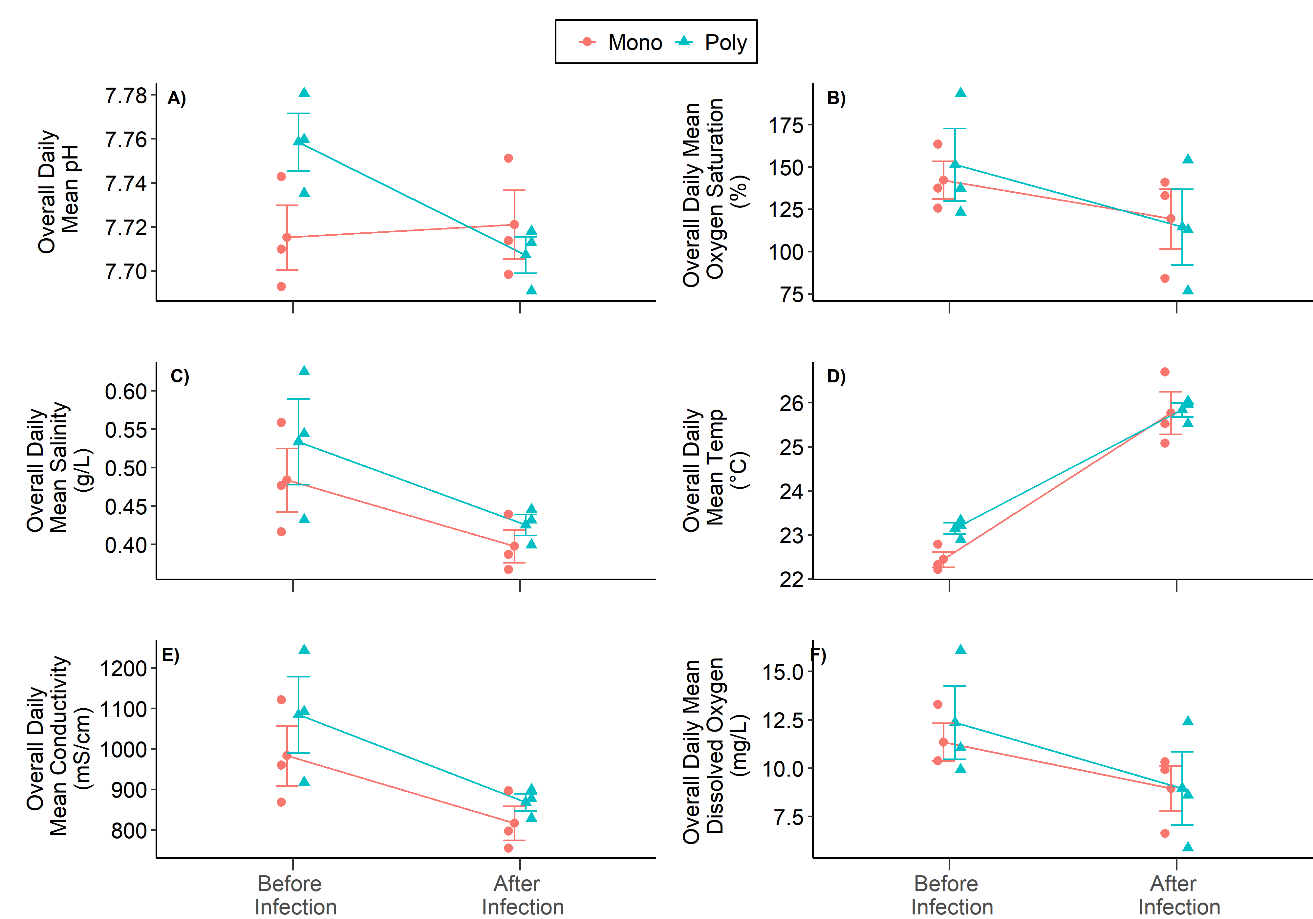


**Figure S4.** Overall mean physical and chemical conditions for ponds by treatment, before and after infection. Vertical bars indicate standard error.

**Table S1**: ANOVA Results for Salinity. pH, and Oxygen Saturation.

|  |  |  | *Salinity* | | | *pH* | | | *Oxygen Saturation* | | |
| --- | --- | --- | --- | --- | --- | --- | --- | --- | --- | --- | --- |
| Effect | *df* |  | MS | *F* | *p* | MS | *F* | *p* | MS | *F* | *p* |
| Treatment | 1 |  | 0.004 | 1.098 | 0.325 | .0006 | 1.231 | 0.329 | 1.23 | 0.007 | 0.936 |
| Period | 1 |  | 0.028 | 6.920 | 0.030 | .002 | 2.939 | 0.161 | 2675.70 | 15.954 | 0.016 |
| Trmt:Period | 1 |  | 0.0004 | 0.089 | 0.774 | .002 | 4.670 | 0.097 | 149.19 | 0.890 | 0.400 |

**Table S2**: ANOVA Results for Temperature, Conductivity, and Dissolved Oxygen concentration.

|  |  |  | *Temperature* | | | *Conductivity* | | | *Dissolved Oxygen* | | |
| --- | --- | --- | --- | --- | --- | --- | --- | --- | --- | --- | --- |
| Effect | *df* |  | MS | *F* | *p* | MS | *F* | *p* | MS | *F* | *p* |
| Treatment | 1 |  | 0.369 | 1.774 | 0.254 | 17627 | 1.422 | 0.267 | 0.053 | 0.057 | 0.823 |
| Period | 1 |  | 27.065 | 130.460 | 0.0003 | 109883 | 8.862 | 0.018 | 25.148 | 27.139 | 0.007 |
| Trmt:Period | 1 |  | 0.300 | 1.446 | 0.295 | 1825 | 0.147 | 0.711 | 0.764 | 0.825 | 0.415 |
